# Antimicrobial efficacy of self-locomotive manganese oxide nanozyme-doped diatom microbubbler on orthodontic brackets in vitro

**DOI:** 10.1186/s12903-023-02739-z

**Published:** 2023-01-20

**Authors:** Hyunsub Kim, Eun-Hyuk Lee, Sang-woo Lee, Yu-Heng Deng, Ho-Beom Kwon, Young-Jun Lim, Hyunjoon Kong, Myung-Joo Kim

**Affiliations:** 1 Department of Prosthodontics, School of Dentistry and Dental Research Institute, Seoul National University, Seoul, 03080 Republic of Korea; 2 Department of Physiology, School of Dentistry and Dental Research Institute, Seoul National University, Seoul, 03080 Republic of Korea; 3 Department of Chemical and Biomolecular Engineering, Carle Illinois College of Medicine, Urbana, IL 61801 USA; 4 Department of Bioengineering, Beckman Institute, Carle Illinois College of Medicine, Carl R. Woese Institute for Genomic Biology, University of Illinois at Urbana-Champaign, Urbana, IL 61801 USA

**Keywords:** Nanozyme, Manganese oxide, Diatom, Biofilm, Bracket

## Abstract

**Background:**

Orthodontic brackets provide a favorable environment for *Streptococcus mutans* biofilm formation, increasing the risk of white spots and dental caries. Manganese oxide (MnO_2_) nanozyme-doped diatom microbubbler (DM) is a recently developed material for biofilm removal. DM can generate oxygen by catalase-mimicking activity in Hydrogen peroxide (H_2_O_2_) solution and move with ejecting oxygen microbubbles to produce a mechanical self-cleansing effect. This study aimed to evaluate the feasibility of DM as a novel bracket cleaner.

**Methods:**

DM was prepared according to the protocol and analyzed using a scanning electron microscope (SEM). We treated *S. mutans* biofilms grown over bracket with phosphate-buffered saline (PBS group), 0.12% chlorhexidine (CHX group), 3% H_2_O_2_ (H_2_O_2_ group), and co-treatment with 3 mg/mL of DM and 3% H_2_O_2_ (DM group). The biofilm removal effect was analyzed using crystal violet assay, and the results were observed using SEM. The viability of *S. mutans* in remaining biofilms was evaluated using confocal laser scanning microscopy (CLSM). Finally, we examined the effect of all materials on mature multispecies biofilms formed on debonded brackets.

**Results:**

Crystal violet assay results revealed that the CHX group removed more biofilms than the control group, and the DM group removed biofilms more effectively than the CHX group (*p* < 0.0001). SEM and CLSM images showed that CHX killed *S. mutans* but failed to remove most biofilms on brackets. However, DM effectively removed biofilms and mature multispecies biofilms on debonded brackets (*p* < 0.0001).

**Conclusions:**

Co-treatment with DM and H_2_O_2_ is effective in removing biofilms on orthodontic brackets compared to conventional antibacterial agents.

## Background

As the number of patients undergoing orthodontic treatment increases [1], the side effects of orthodontic treatment have also increased [2, 3]. *Streptococcus mutans* is is a major contributor of biofilm formation associated with dental caries [4]. The placement of orthodontic appliances provides an environment for the proliferation of plaque-producing bacteria such as *S. mutans* [5–7]. Of the many orthodontic appliances, brackets play a significant role in enamel demineralization because they are difficult to clean due to their complex design and remain attached to the dentition throughout orthodontic treatment [8]. Therefore, it is important to maintain good oral hygiene during orthodontic treatment.

Due to the complex design of orthodontic brackets, mechanical methods such as brushing cannot ensure complete plaque removal [6, 9]. To overcome the limitations of mechanical cleaning methods, chemical antimicrobial agents, such as chlorhexidine (CHX) and hydrogen peroxide (H_2_O_2_), have been used for plaque control [10–12]. However, bacteria residing in biofilms have higher resistance to antibiotics and disinfectants than planktonic cells because the matrix produced by extracellular polymeric substances (EPS) limits the transport of antibacterial agents and neutralizes them chemically [13, 14]. It is necessary to study new cleaning techniques that effectively remove biofilms on orthodontic brackets. Therefore, many previous studies have investigated materials and methods for more effective plaque removal from brackets [10, 12, 15–17].

Manganese oxide (MnO_2_) nanozyme-doped diatom microbubbler (DM) is recently developed as an active cleaning agent by loading MnO_2_ nanozyme sheets on fossilized *Aulacoseira* diatom particles [18]. DM is a hollow cylinder-shaped microparticle with many pores on the surface. In the H_2_O_2_ solution, the catalase-mimicking activity of the MnO_2_ nanozyme sheet of the DM rapidly decomposes H_2_O_2_ and generates oxygen. With the propelling force of oxygen gas, DM moves randomly, destroys and penetrates the matrix in biofilms, and continuously generates microbubbles. DM can effectively remove biofilms from complex structures in confined spaces [18], and be considered a novel cleaning agent for decontamination of biofilms from orthodontic brackets.

There has been no study so far on cleaning brackets using DM. The present in vitro study aimed to evaluate the feasibility of using DM as a novel bracket cleaner. It compares the ability of DM to remove *S. mutans* biofilms formed on metal brackets with that of conventional antibacterial agents, such as CHX and H_2_O_2_. It also evaluates the effect of DM in removing mature biofilms formed over a long period on brackets in the oral cavity of patients.

## Methods

### Preparation of the DMs

**Fig. 1:**
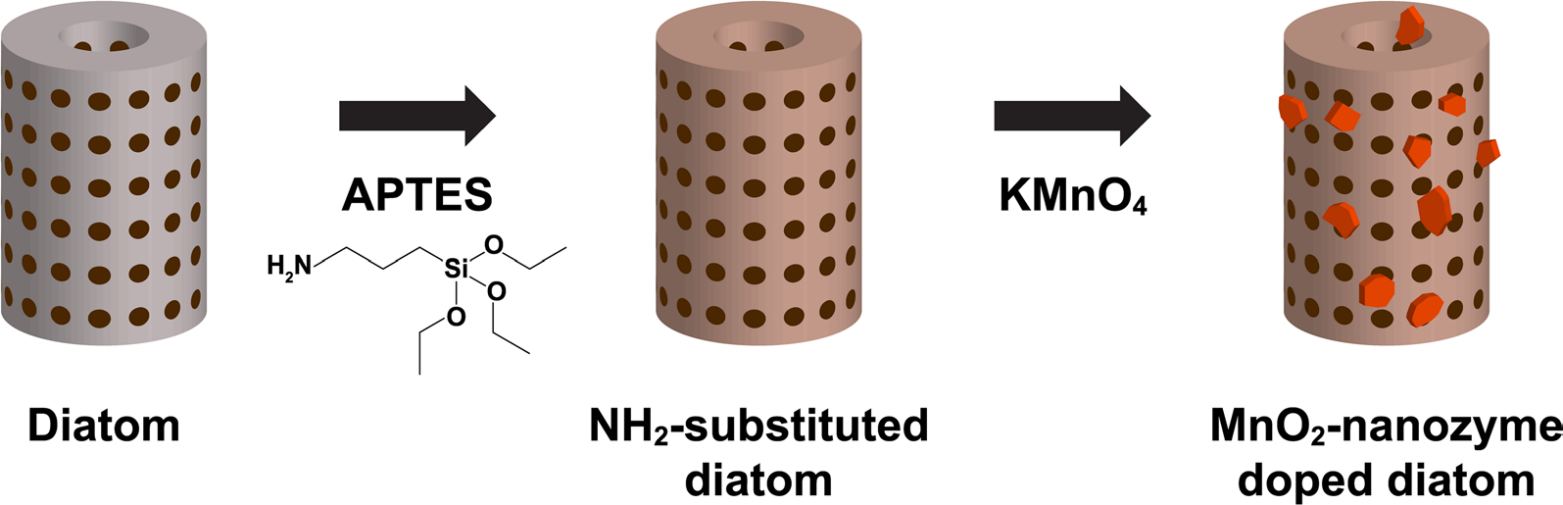
Schematic illustration of fabrication steps for MnO_2_ nanozyme-doped diatom microbubbler

For DM preparation, 2 g of diatom particles, 60 mL of toluene, and 0.6 mL of distilled water were added to a three-necked round-bottom flask equipped with a thermometer, a reflux condenser, and an N_2_ gas tube according to the protocol of the previous study (Fig. 1) [18]. The mixture was stirred for 2 h at room temperature; 3.4 mL of (3-aminopropyl) triethoxysilane (APTES; Sigma-Aldrich, St. Louis, MO, USA) was added and refluxed for 6 h at 60 °C. The mixture was cooled and washed with toluene, 2-propanol, and distilled water. After drying in a vacuum desiccator for 2 days, 0.1 g of amine-substituted diatom particles were added to 1 mL of 50 mM potassium permanganate (KMnO4; Sigma-Aldrich, St. Louis, MO, USA) solution and sonicated for 30 min at room temperature. Next, the particles were washed with distilled water and ethanol and dried in an oven for 1 day at 60 °C.

### Physicochemical characterization of DMs

Scanning electron microscope (SEM) images were taken with Apreo S (Thermo Fisher Scientific, Waltham, MA, USA) at 15,000 and 50,000 times magnification at 10.0 kV to observe the morphology of prepared DMs. The elemental analyses were performed using an energy dispersive spectrometer (EDS) coupled with SEM at 20.0 kV to confirm that the MnO_2_ nanozyme sheets were well-doped on the surface of the diatom particles.

### Biofilm formation on orthodontic brackets

Sterile lower incisor brackets (Empower 2; American Orthodontics, Sheboygan, WI, USA) were placed in a 96-well tissue culture plate and inoculated with 200 μL of *S. mutans* (ATCC 25175) suspension brain–heart infusion with 1% sucrose at a concentration of 10^6^ CFU/mL. Then the plate was incubated anaerobically for 24 h at 37 °C.

### Crystal violet assay

A total of 50 sterile brackets were prepared and randomly assigned to 5 groups for crystal violet assay (n = 10). Of these, 10 specimens were placed in 200 mL of sterile broth as a negative control group, and the specimens of the other 4 groups were incubated with *S. mutans* suspension as previously described to form biofilms. After biofilm formation, the orthodontic brackets were gently washed with phosphate-buffered saline (PBS, pH = 7.4) and transferred to a new 96-well tissue culture plate. Next, orthodontic brackets were treated either with PBS (PBS group), 0.12% (w/v) CHX (Bukwang Pharmaceutical Co., Ltd, Seoul, Korea) (CHX group), 3% (v/v) H_2_O_2_ (H_2_O_2_; Sigma-Aldrich, St. Louis, MO) (H_2_O_2_ group), or co-treatment with 3 mg/mL of DM and 3% H_2_O_2_ (DM group) for 2 min.

After removing non-adherent cells by washing with PBS, the remaining biofilms on the brackets were quantified using a crystal violet assay [19]. The brackets were stained with 1 mL of 1% (w/v) crystal violet solution (Junsei Chemical, Tokyo, Japan) for 10 min and washed with PBS thrice. The crystal violet dye in the remaining biofilms was released using 95% ethanol. Subsequently, 200 μL of the solution was transferred to a microtiter plate, and the optical density (OD) of the dissolved crystal violet dye was measured using a microplate reader (Epoch 2; Bio-Tek Instruments, Winooski, VT, USA) at 570 nm.

### SEM analysis

SEM (Apreo S; Thermo Fisher Scientific, Waltham, MA, USA) was used to visualize the remaining biofilms on the brackets after each treatment. The non-adherent cells were removed by washing with PBS, and the brackets were fixed for 4 h with 1 mL of 4% paraformaldehyde (Biosesang, Seongnam, Korea) and washed with PBS for 15 min thrice. Next, the specimens were dehydrated in successively increasing concentrations of ethanol for 15 min (70%, 80%, 90%, 95%, and 100%). Subsequently, the resin specimens were treated in 1 mL of 100% hexamethydilazane (HMDS; Sigma-Aldrich, St. Louis, MO, USA) for 20 min, dried completely, and coated with platinum. The biofilms on the brackets were visualized using SEM at a voltage of 10 kV.

### Staining and visualization of biofilms by CLSM

The biofilms on the brackets were washed with PBS and stained with the molecular probes’ Live/Dead BacLight viability kit comprising SYTO-9 and propidium iodide (Invitrogen, Eugene, OR, USA) to visualize the viability of the remaining biofilm after each treatment.^12)^ The biofilms were incubated with the staining solution containing SYTO-9 and propidium iodide for 20 min in a dark cabinet. The brackets were washed with PBS thrice and placed upside-down on glass-bottomed confocal dishes (SPL Life Science, Kyong‐Gi, Korea) with BacLight mounting oil (Thermo Fisher Scientific, Waltham, MA, USA). The stained biofilms were immediately examined using CLSM (LSM700; Carl Zeiss, Oberkochen, Germany).

### Biofilm removal assay on contaminated orthodontic brackets debonded from patients

A total of 32 brackets were collected from orthodontic patients on the day of debonding and randomly assigned to 4 groups (n = 8). The brackets were gently washed twice with PBS and stained with 100 µg/mL FITC-conjugated concanavalin A (Sigma-Aldrich, St. Louis, MO, USA) for 20 min in a dark cabinet to label microorganisms on the brackets. After washing with PBS twice, stained samples were imaged using a fluorescence imaging system (FOBI; Neoscience, Suwon, Korea). The brackets were placed in a 96-well tissue culture plate and treated for each group. After washing with PBS thrice, images of the brackets were taken using the fluorescence imaging system. Stained areas of the brackets “before treatment” and “after treatment” were measured using an image analyzing software program (ImageJ; National Institutes of Health, Bethesda, MD, USA). This study was approved by the Ethics Committee of Seoul National University Dental Hospital (ERI22013).

### Statistical analysis

The Shapiro–Wilk normality test revealed that the data were normally distributed (*p* > 0.05), and homogeneity of variance was checked by Levene’s test (α = 0.05). The quantitative data of the crystal violet assay were statistically analyzed using a 1-way analysis of variance (ANOVA) and Tukey’s multiple comparison test (α = 0.05). A 2-way repeated-measures ANOVA test was performed to analyze the plaque removal assay on brackets debonded from patients (α = 0.05). The Bonferroni method was used for pairwise comparisons of the areas of stained biofilms on the brackets before and after treatment (α = 0.05). Tukey’s multiple comparison test was performed for comparisons among groups (α = 0.05). Statistical analyses were performed using a statistical program (Prism 9; GraphPad, San Diego, CA, USA).

## Results

### Physicochemical characterization of DMs

**Fig. 2:**
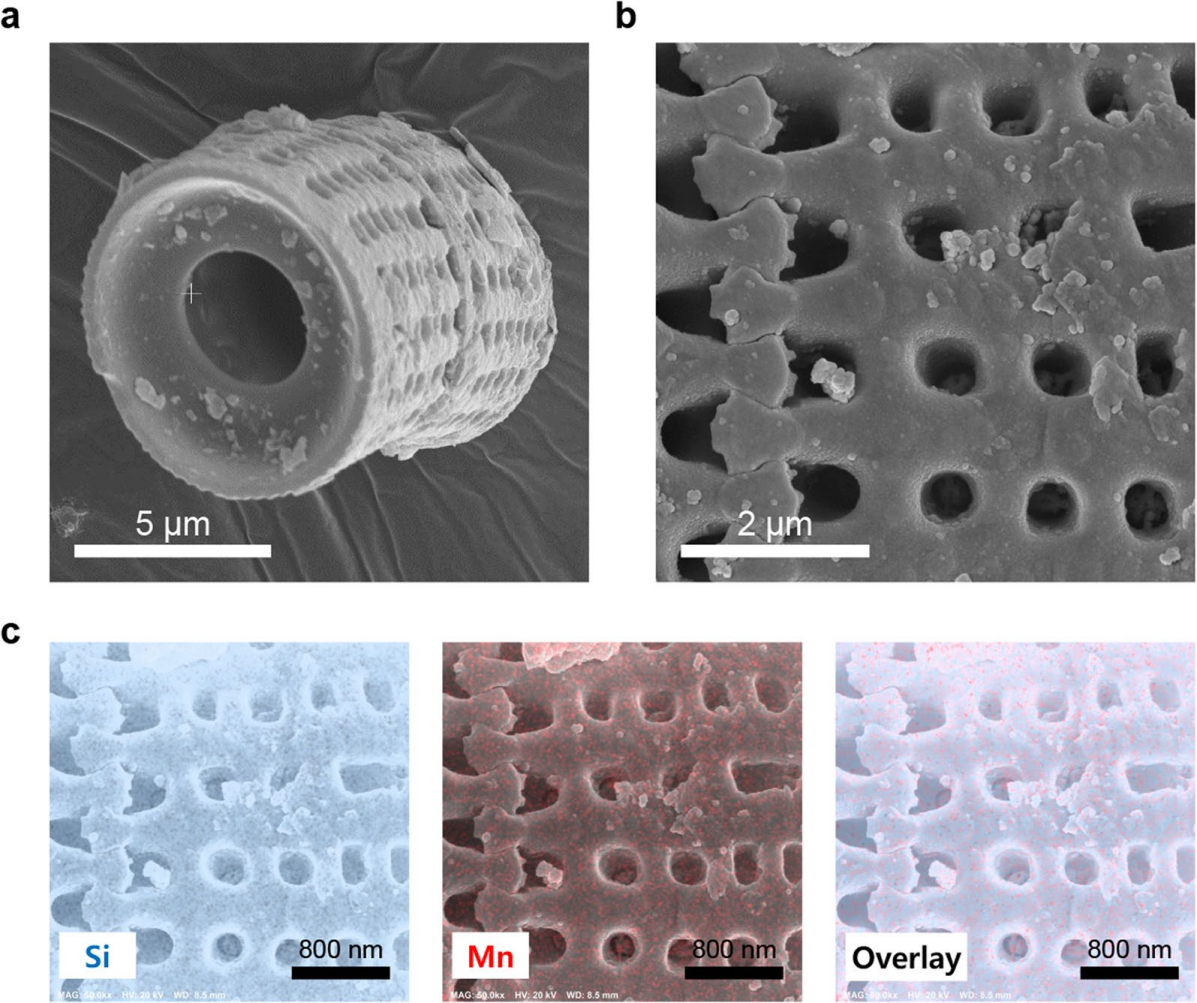
Scanning electron microscopy images and element mapping images of the fabricated MnO_2_ nanozyme-doped diatom microbubbler. **a** Scanning electron microscopy image of diatom microbubbler (magnification: 15,000 times, white scale bar = 5 μm). **b** Scanning electron microscopy image shows pores on the surface of the diatom microbubbler (magnification: 50,000 times, white scale bar = 2 μm). **c** Element mapping images showing a homogenous distribution of MnO_2_ nanozyme sheets on the diatom microbubbler (black scale bar = 800 nm)

The diatom particles used in this study had many pores on the surface in the form of a hollow cylinder (Fig. 2a, b). The element mapping images confirmed that MnO_2_ nanozyme sheets were uniformly loaded onto the DM surface (Fig. 2c).

### Crystal violet assay

**Fig. 3:**
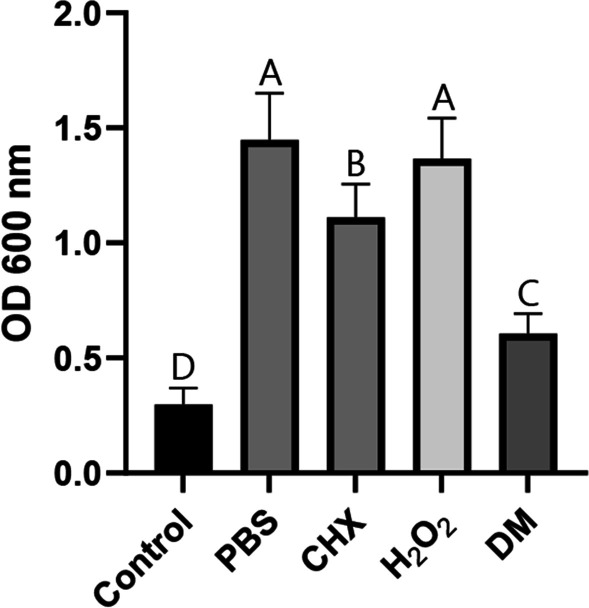
*Streptococcus mutans* biofilm removal effect evaluated using crystal violet analysis. Data are expressed as mean value ± standard deviation. Data were analyzed using 1-way ANOVA with Tukey’s multiple comparison tests (*p* < 0.05). Different letters denote statistical differences between groups

The results of the crystal violet assay that evaluated the remaining biofilms on the bracket after each decontamination treatment are shown in Fig. 3. The CHX group showed a significantly lower value than the PBS group (*p* < 0.0001), and the DM group had a lower value than the CHX group (*p* < 0.0001). The H_2_O_2_ group did not show a statistically significant difference from the PBS group (*p* > 0.05).

### SEM analysis

**Fig. 4:**
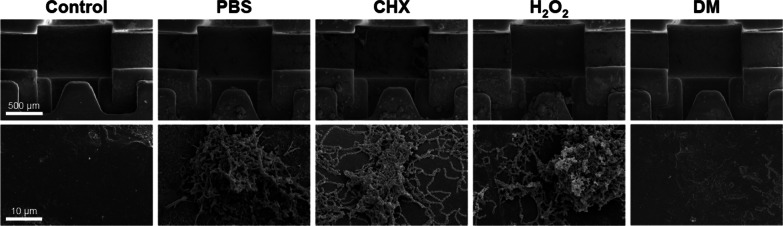
Scanning electron microscopy images of orthodontic brackets after each biofilm removal treatment. The images in the upper row were taken at 200 × (scale bar = 500 μm), and the images in the lower row were taken at 10,000 × (scale bar = 10 μm)

SEM images for visually evaluating the remaining biofilms on the brackets are presented in Fig. 4. The cluster of *S. mutans* remains in the CHX and H_2_O_2_ groups as in the PBS group. However, in the DM group, there were very few remaining *S. mutans*, and we observed only damaged or fragmented residues.

### Staining and visualization of biofilms by CLSM

**Fig. 5:**
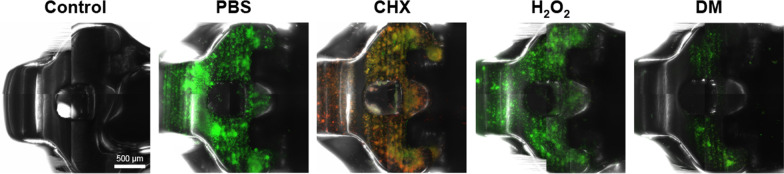
Confocal laser scanning microscopy images of remaining biofilms on orthodontic brackets after each biofilm removal treatment. Dual-staining method using SYTO-9 and propidium iodide was used to evaluate the viability of remaining *Streptococcus mutans* after each decontamination treatment. SYTO-9 stained all bacterial cells, while propidium iodide stained dead bacterial cells. The white scale bar represents 500 µm

According to the CLSM images shown in Fig. 5, a large proportion of *S. mutans* in the remaining biofilms were stained with propidium iodide in the CHX group. Only a few cells were left in the DM group compared to other groups.

### Biofilm removal assay on contaminated orthodontic brackets debonded from patients

**Fig. 6:**
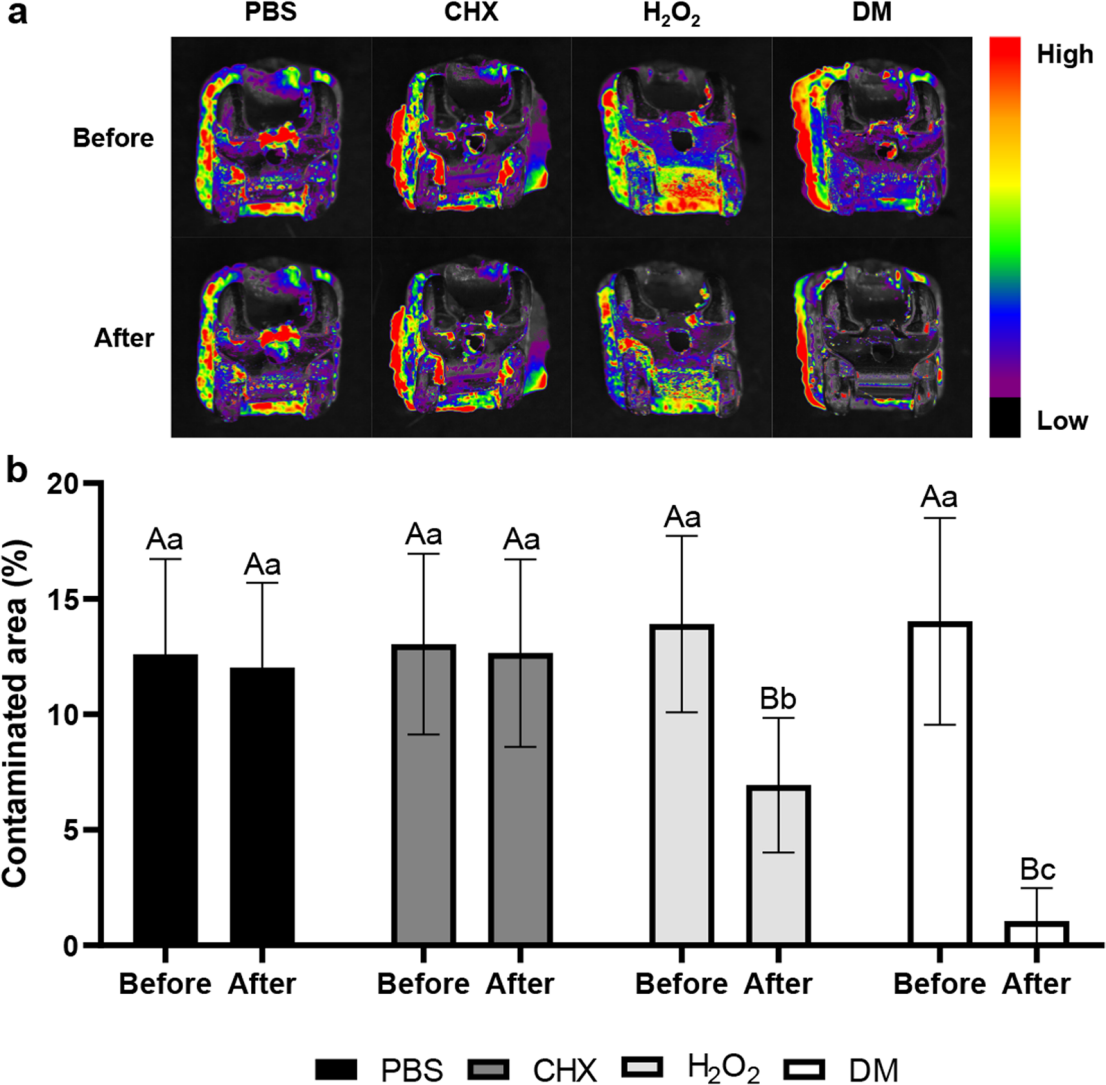
Plaque removal assay of contaminated brackets debonded from patients. **a** Relative fluorescence heatmap for remaining biofilms stained with FITC-conjugated concanavalin A before and after each treatment. **b** Relative biofilm-contaminated areas of brackets before and after treatment are quantified. Different uppercase letters denote statistical differences in the stained area before and after treatment within the same group (*p* < 0.05). Different lowercase letters denote statistical differences between tested groups at the same time point (*p* < 0.05)

The biofilms of the brackets used by the patients were stained using FITC-conjugated concanavalin A; the stained areas before and after each treatment were comparatively analyzed. The 2-way repeated-measures ANOVA showed the significance of groups, treatment, and interactions among these factors in the areas of stained biofilms on debonded brackets (Table 1). There was no significant difference in the stained area before and after treatment in the PBS and CHX groups (Fig. 6). However, the stained areas of the H_2_O_2_ and DM groups showed a statistically significant reduction after each treatment (*p* < 0.0001). After treatment, the DM group had fewer stained areas than the H_2_O_2_ group (*p* = 0.0113).

**Table 1:** Results of 2-way repeated measures ANOVA for measured area of stained biofilms on debonded orthodontic brackets

Source	Type III sum of square	df	Mean square	F	Sig
Group (G)	432.4	3	144.1	46.75	< 0.0001
Treatment (T)	438.0	1	438.0	142.1	< 0.0001
G × T	275.6	3	91.88	3.868	0.0197
Subject	665.0	28	23.75	7.704	< 0.0001
Residual	86.33	28	3.083

## Discussion

White spots and dental caries occurring during orthodontic treatment are problematic for patients and orthodontists [20]. *S. mutans*, the bacterium used in this study, lowers the pH and tilts the demineralization-remineralization equilibrium toward mineral loss, causing white spots and dental caries [4, 21]. Studies show raised *S. mutans* levels in the oral cavity and increased risk of dental caries after bonding orthodontic brackets [22, 23].

Effective plaque removal using mechanical cleaning methods can present challenges due to the complex design of orthodontic brackets [6]. Therefore, chemical methods should be used as an assistant [17]. CHX has been used as a conventional chemical therapeutic agent for plaque control [10, 11]. A previous study reported that CHX reduced the viability of *S. mutans* on brackets [17]. The present study showed that CHX effectively removed *S. mutans* biofilms on brackets (Fig. 3). The SYTO-9 from the Live/Dead BacLight viability kit labels all remaining cells (with intact membranes and damaged membranes). In contrast, propidium iodide penetrates only bacteria with damaged membranes [12, 24]. CLSM images in which a large proportion of the bacteria on the brackets were treated with CHX and stained with propidium iodide showed that CHX could kill *S. mutans* (Fig. 5). However, CHX did not effectively remove mature multispecies biofilms on the brackets used by the patients (Fig. 6), and CHX efficacy reduced against bacteria in the biofilms [13, 14]. The EPS matrix of biofilms resists chemical antimicrobial agents, and the compact matrix inhibits the diffusion of solutes [25].

The amounts of *S. mutans* biofilms were not significantly different from the PBS group when using the H_2_O_2_ solution alone (Fig. 3). However, mature multispecies biofilms on debonded brackets significantly reduced by H_2_O_2_ treatment (Fig. 6). One mechanism of the antibacterial action of H_2_O_2_ is the liberation of oxygen by catalase [26]. Bacterial catalase enzymes decompose H_2_O_2_ and generate oxygen bubbles that break biofilms [27]. Since *S. mutans* cannot produce catalase [28, 29], biofilm removal by this mechanism did not occur (Fig. 3). However, several types of microorganisms in the oral cavity release catalase, and saliva contains catalase derived from bacteria [30]. Therefore, H_2_O_2_ treatment of multispecies biofilms on debonded brackets generated oxygen bubbles and significantly reduced stained biofilms. The cleaning effect of H_2_O_2_ treatment alone was affected by biofilm composition.

This study evaluated DM as a novel orthodontic bracket cleaner that can overcome the limitations of existing chemical antimicrobial agents. DM, produced by doping MnO_2_ nanozyme sheets on fossilized *Aulacoseira* diatom, is a microparticle (approximately 10 µm in diameter and 18 µm in length) developed to remove biofilms (Fig. 2a) [18], and is unfamiliar to the dental field. DM has the shape of a hollow cylinder, and the wall has a large number of pores with an average diameter of 500 nm (Fig. 2b). Oxygen can be generated by the catalase-mimicking activity of MnO_2_ in an H_2_O_2_ solution. The oxygen collected inside the cylinder creates pressure, and the DM particle moves randomly by ejecting oxygen microbubbles [18]. Reports show that the migration rate of DM in 3% H_2_O_2_ is 60 µm/s [18]. Using this principle, DM destroys biofilms, penetrates them, and moves randomly while continuously generating oxygen gas. H_2_O_2_ can also diffuse into destroyed biofilms.

As DM mechanically destroys the structure of biofilms, it can overcome the limitations of conventional chemical antimicrobial agents. Although CHX killed bacteria (Fig. 5), it did not effectively remove mature multispecies biofilms (Fig. 6), and H_2_O_2_ was ineffective in removing *S. mutans* biofilms (Fig. 3). However, co-treatment with DM and H_2_O_2_ effectively removed *S. mutans* biofilms and mature multispecies biofilms from brackets compared to CHX and H_2_O_2_ (Fig. 3 and 6). DM particles can infiltrate biofilms and continuously produce oxygen microbubbles that merge and burst to deform and fracture the biofilms; therefore, co-treatment with H_2_O_2_ and DM removed biofilms more effectively than H_2_O_2_ treatment alone (Fig. 6) [18]. The SEM image of the bracket in the DM group showed cluster structure destruction of the biofilms (unlike other groups), leaving only very few *S. mutans* and damaged or fragmented residues (Fig. 4).

CLSM images of the DM group showed fewer stained bacteria than other groups (Fig. 5). Although CHX killed *S. mutans*, several dead cells and biofilm structures remained on the bracket (Fig. 5); the remaining biofilm structure and the extracellular matrix promote microbial adhesion and cohesion [14], and provide a three-dimensional scaffold for biofilm development [31, 32]. EPS of cariogenic biofilms formed by *S. mutans* is an essential virulence factor related to dental caries [33, 34]. Although CHX kills *S. mutans*, it cannot remove the structure of most biofilms, so the risk of additional biofilm development and dental caries progression remains. However, DM can effectively reduce this potential risk by the mechanical removal of biofilms.

Reports show that DM can effectively remove biofilms in confined spaces [18]. Therefore, biofilms existing in narrow or hidden spaces of brackets that cannot be removed using conventional mechanical cleaning methods, such as brushing, can be effectively removed by DM. Moreover, unlike conventional chemical agents, DM removed biofilms from the brackets and damaged the biofilm structure to break bacterial resistance and defense functions, enabling more effective decontamination. Although this in vitro study showed that DM could be a novel therapeutic agent for the decontamination of orthodontic brackets, further research on practical methods for using DM in actual clinical situations should also be developed and optimized. As shown in previously studied oral cleaning devices [35, 36], an oral tray can be used to create a confined space around teeth and brackets enabling DM to remove biofilms more effectively. It should be noted that our study cannot fully predict the physicochemical behavior of DM in human oral cavity. Therefore, care should be taken in translating the results of this in vitro study to an in vivo situation. However, biocompatibility of DM in has been extensively examined in a previous study by repetitively co-treatment of DM and H_2_O_2_ on tongue mucosa, resulting in no significant tissue damage or inflammation [37]. In addition, when MG63 cells were exposed to DM for 24 h, no significant cytotoxicity was observed up to concentration of 10 mg/mL [37]. However, in order for DM to be applied in the oral cavity in clinical setting, further studies are required for biocompatibility of DM on various oral tissues including gingiva, palate, and buccal mucosa.

## Conclusions

- Co-treatment with H_2_O_2_ and DM effectively removed *S. mutans* biofilms and multispecies biofilms on the bracket surfaces, showing the possibility of being used as a novel bracket cleaner.
- Co-treatment with H_2_O_2_ and DM removed *S. mutans* biofilms more effectively than CHX, and the removal effect of mature multispecies biofilms was also significantly superior to that of H_2_O_2_ treatment alone.
- CHX killed *S. mutans* on the brackets and effectively removed *S. mutans* biofilms, but not mature multispecies biofilms formed on brackets in the oral cavity for a prolonged period.
- H_2_O_2_ did not effectively remove *S. mutans* biofilms but significantly reduced multispecies biofilms on debonded brackets.

## Data Availability

The datasets used and/or analysed during the current study are available from the corresponding author on reasonable request.

## Abbreviations

DM: Manganese oxide nanozyme-doped diatom microbubbler
MnO_2_: Manganese oxide
SEM: Scanning electron microscope
H_2_O_2_: Hydrogen peroxide
CHX: Chlorhexidine
CLSM: Confocal laser scanning microscopy
EPS: Extracellular polymeric substances
EDS: Energy dispersive spectrometer
PBS: Phosphate-buffered saline
OD: Optical density

## References

[1] Nattrass C, Sandy J (1995). Adult orthodontics—a review. Br J Orthod.

[2] Shungin D, Olsson AI, Persson M (2010). Orthodontic treatment-related white spot lesions: a 14-year prospective quantitative follow-up, including bonding material assessment. Am J Orthod Dentofac Orthop.

[3] Mitchell L (1992). Decalcification during orthodontic treatment with fixed appliances—an overview. Br J Orthod.

[4] Beighton D (2005). The complex oral microflora of high-risk individuals and groups and its role in the caries process. Community Dent Oral Epidemiol.

[5] Diamanti-Kipioti A, Gusberti FA, Lang NP (1987). Clinical and microbiological effects of fixed orthodontic appliances. J Clin Periodontol.

[6] Pandis N, Papaioannou W, Kontou E, Nakou M, Makou M, Eliades T (2010). Salivary *Streptococcus mutans* levels in patients with conventional and self-ligating brackets. Eur J Orthod.

[7] Rosenbloom RG, Tinanoff N (1991). Salivary *Streptococcus mutans* levels in patients before, during, and after orthodontic treatment. Am J Orthod Dentofac Orthop.

[8] Ahn SJ, Lee SJ, Lim BS, Nahm DS (2007). Quantitative determination of adhesion patterns of cariogenic streptococci to various orthodontic brackets. Am J Orthod Dentofac Orthop.

[9] Hägg U, Kaveewatcharanont P, Samaranayake Y, Samaranayake L (2004). The effect of fixed orthodontic appliances on the oral carriage of *Candida* species and *Enterobacteriaceae*. Eur J Orthod.

[10] Panariello BH, Cavichioli EA, Sochacki SF, Junior LGG, Duarte S (2022). Effect of blue light plus chlorhexidine therapy on *Streptococcus mutans* biofilm and its regrowth in an in vitro orthodontic model. Am J Orthod Dentofac Orthop.

[11] Mathur S, Mathur T, Srivastava R, Khatri R (2011). Chlorhexidine: the gold standard in chemical plaque control. Natl J Physiol Pharm Pharmacol.

[12] Chen Y, Wong RW, Seneviratne CJ, Hägg U, McGrath C, Samaranayake LP (2011). Comparison of the antimicrobial activity of Listerine and Corsodyl on orthodontic brackets in vitro. Am J Orthod Dentofac Orthop.

[13] Voo ZX, Khan M, Xu Q, Narayanan K, Ng BW, Ahmad RB (2016). Antimicrobial coatings against biofilm formation: the unexpected balance between antifouling and bactericidal behavior. Polym Chem.

[14] Flemming H-C, Wingender J (2010). The biofilm matrix. Nat Rev Microbiol.

[15] Chen Y, Wong RW, Seneviratne CJ, Hägg U, McGrath C, Samaranayake LP (2011). The antimicrobial efficacy of Fructus mume extract on orthodontic bracket: a monospecies-biofilm model study in vitro. Arch Oral Biol.

[16] Mukumoto M, Ohshima T, Ozaki M, Konishi H, Maeda N, Nakamura Y (2012). Effect of microbubbled water on the removal of a biofilm attached to orthodontic appliances—an in vitro study—. Dent Mater J.

[17] Dias AP, Paschoal MAB, Diniz RS, Lage LM, Gonçalves LM (2018). Antimicrobial action of chlorhexidine digluconate in self-ligating and conventional metal brackets infected with *Streptococcus mutans* biofilm. Clin Cosmet Investig Dent.

[18] Seo Y, Leong J, Park JD, Hong YT, Chu SH, Park C (2018). Diatom microbubbler for active biofilm removal in confined spaces. ACS Appl Mater Interfaces.

[19] Ho CS, Ming Y, Foong KW, Rosa V, Thuyen T, Seneviratne CJ (2017). *Streptococcus mutans* forms xylitol-resistant biofilm on excess adhesive flash in novel ex-vivo orthodontic bracket model. Am J Orthod Dentofac Orthop.

[20] Richter AE, Arruda AO, Peters MC, Sohn W (2011). Incidence of caries lesions among patients treated with comprehensive orthodontics. Am J Orthod Dentofac Orthop.

[21] Featherstone JD (2003). The caries balance: contributing factors and early detection. J Calif Dent Assoc.

[22] Scheie AA, Arneberg P, Krogstad O (1984). Effects of orthodontic treatment on prevalence of *Streptococcus mutans* in plaque and saliva. Eur J Oral Sci.

[23] Lundström F, Krasse B (1987). Caries incidence in orthodontic patients with high levels of *Streptococcus mutans*. Eur J Oral Sci.

[24] Williams S, Hong Y, Danavall D, Howard-Jones M, Gibson D, Frischer M (1998). Distinguishing between living and nonliving bacteria: evaluation of the vital stain propidium iodide and its combined use with molecular probes in aquatic samples. J Microbiol Methods.

[25] Hope C, Wilson M (2004). Analysis of the effects of chlorhexidine on oral biofilm vitality and structure based on viability profiling and an indicator of membrane integrity. Antimicrob Agents Chemother.

[26] Marshall MV, Cancro LP, Fischman SL (1995). Hydrogen peroxide: a review of its use in dentistry. J Periodontol.

[27] Clemente A, Alba-Patiño A, Rojo-Molinero E, Russell SM, Borges M, Oliver A (2020). Rapid Detection of *Pseudomonas aeruginosa* biofilms via enzymatic liquefaction of respiratory samples. ACS Sens.

[28] Jakubovics NS, Gill SR, Vickerman MM, Kolenbrander PE (2008). Role of hydrogen peroxide in competition and cooperation between *Streptococcus gordonii* and *Actinomyces naeslundii*. FEMS Microbiol Ecol.

[29] Baldeck JD, Marquis RE (2008). Targets for hydrogen-peroxide-induced damage to suspension and biofilm cells of *Streptococcus mutans*. Can J Microbiol.

[30] Sidaway D (1978). A microbiological study of dental calculus: I. The microbial flora of mature calculus. J Periodontal Res.

[31] Stewart PS, Franklin MJ (2008). Physiological heterogeneity in biofilms. Nat Rev Microbiol.

[32] Mann EE, Wozniak DJ (2012). Pseudomonas biofilm matrix composition and niche biology. FEMS Microbiol Rev.

[33] Klein MI, Hwang G, Santos PH, Campanella OH, Koo H (2015). *Streptococcus mutans*-derived extracellular matrix in cariogenic oral biofilms. Front Cell Infect Microbiol.

[34] Mattos-Graner R, Smith D, King W, Mayer M (2000). Water-insoluble glucan synthesis by mutans streptococcal strains correlates with caries incidence in 12-to 30-month-old children. J Dent Res.

[35] Lee JH, Jung KW, Kim HK, Koo KT, Kim SH (2016). Evaluation of effectiveness of vacuum oral cleaner developed for patients with limited mobility. Taehan Chikkwa Uisa Hyophoe Chi.

[36] Howlin RP, Fabbri S, Offin DG, Symonds N, Kiang KS, Knee RJ (2015). Removal of dental biofilms with an ultrasonically activated water stream. J Dent Res.

[37] Lee EH, Lee SW, Seo Y, Deng YH, Lim YJ, Kwon HB (2022). Manganese oxide nanozyme-doped diatom for safe and efficient treatment of peri-implantitis. ACS Appl Mater Interfaces.

